# Supplementary material for: Exploring Micromonospora as Phocoenamicins Producers
Source: Mar Drugs. 2022 Dec 7;20(12):769. doi: 10.3390/md20120769 (PMC9782249; doi:10.3390/md20120769)
Supplement: Supplementary file 1 [file marinedrugs-20-00769-s001.zip › marinedrugs-2013771-supplementary.pdf]

Exploring *Micromonospora* as Phocoenamicins Producers

Maria Kokkini*, Cristina González Heredia, Daniel Oves-Costales, Mercedes de la Cruz, Jesús Martín, Francisca Vicente, Olga Genilloud and Fernando Reyes*

Fundación MEDINA, Centro de Excelencia en Investigación de Medicamentos Innovadores en Andalucía. Parque Tecnológico Ciencias de la Salud. Avda. del Conocimiento 34, 18016 Armilla, Granada, Spain;

* Correspondence: maria.kokkini@medinaandalucia.es (M.K.); fernando.reyes@medinaandalucia.es (F.R.) Tel.: +34-958-993-965 (F.R.)

Contents

Table S1. Composition of the 10 culture media used in the OSMAC approach.

Figure S1. Total Ion Chromatogram (TIC) of the crude extract from the strain CA-214658 grown in RAM2-P V2 medium.

Figure S2. The ESI-TOF spectrum of phocoenamicin.

Figure S3. The ESI-TOF spectrum of phocoenamicin B.

Figure S4. The ESI-TOF spectrum of phocoenamicin C.

Figure S5. The boxplot and density plot showing the normalization result in MetaboAnalyst 5.0 after sample normalization, data transformation and scaling of the preprocessed LC-HRMS data obtained from the 270 extracts.

Figure S6. The boxplot and density plot showing the normalization result in MetaboAnalyst 5.0 after sample normalization, data transformation and scaling of the preprocessed LC-HRMS data obtained from the 27 extracts in RAM2-P V2 medium.

Figure S7. PLS-DA 2D Score plots of the 270 extracts for the parameters (a) different strains, (b) taxonomy species, (c) geographic origin and (d) ecology. All four models resulted in overfitting and lack of validation.

Figure S8. (a) Cross-validation test (CV) with values of R_2=0.859 and Q_2=0.841 and (b) permutation test with p-value < 0.01 of the PLS-DA analysis of the 270 extracts grouped by culture medium that validated its significance.

Figure S9. The classical molecular network (GNPS) generated from the extracts of the 27 strains cultured in RAM2-P V2 medium. The nodes are coloured according to the geographic origin of the 27 strains.

Table S2. Average % inhibition of the 270 extracts against MRSA MB5393, *M. bovis* BCG and *M. tuberculosis* H37Ra.

Table S1. Composition of the 10 culture media used in the OSMAC approach.

Culture medium	Carbon source	Nitrogen source	Trace elements and additional components
**APM9**	Glucose (SIGMA G8270)	Soybean Flour (SIGMA S9633)	$CoCl_2 \cdot 6H_2O$ (SIGMA C8661)
	Soluble starch from potato (PANREAC 121096)	-	$CaCO_3$ (ACROS ORGANICS 450680010)
**DEF-15**	Sucrose (FISHER S8600/70)	NH_4Cl (PANREAC 141121)	Na_2SO_4 (MERCK 1.06649)
	-	-	K_2HPO_4 (MERCK 5101)
	-	-	$MgCl_2 \cdot 6H_2O$ (MERCK 5833)
	-	-	NaCl (MERCK 1.06404)
	-	-	$CaCO_3$ (ACROS ORGANICS 450680010)
	-	-	$MnCl_2 \cdot 4H_2O$; $ZnCl_2$; $FeCl_2 \cdot 4H_2O$; NaCl
**DEF-15-S**	Sucrose (FISHER S8600/70)	NH_4Cl (PANREAC 141121)	Na_2SO_4 (MERCK 1.06649)
	Soluble starch from potato (PANREAC 121096)	-	K_2HPO_4 (MERCK 5101)
	-	-	$MgCl_2 \cdot 6H_2O$ (MERCK 5833)
	-	-	NaCl (MERCK 1.06404)
	-	-	$CaCO_3$ (ACROS ORGANICS 450680010)
	-	-	$MnCl_2 \cdot 4H_2O$; $ZnCl_2$; $FeCl_2 \cdot 4H_2O$; NaCl
**DNPM**	Dextrin from corn Type I (SIGMA D2006)	N-Z Soy BL (SIGMA P6713)	MOPS (FISHER BP 308)
	-	Bacto yeast extract (DIFCO 212750)	-
**FPY-12**	Fructose (PANREAC 142728)	Bacto peptone (DIFCO 211677)	$FeSO_4 \cdot 7H_2O$; $ZnSO_4 \cdot 7H_2O$;

	Glucose (SIGMA G8270)	Amicase (SIGMA A2427)	$MnSO_4 \cdot H_2O$; $CuSO_4 \cdot 5H_2O$; $CoCl_2 \cdot 6\ H_2O$
	Maltose (MERCK 1.05910)	-	-
**FPY-2**	Fructose (PANREAC 142728)	Bacto peptone (DIFCO 211677)	$FeSO_4 \cdot 7H_2O$; $ZnSO_4 \cdot 7H_2O$;
	Sucrose (FISHER S8600/70)	Bacto yeast extract (DIFCO 212750)	$MnSO_4 \cdot H_2O$; $CuSO_4 \cdot 5H_2O$; $CoCl_2 \cdot 6\ H_2O$
	Maltose (MERCK 1.05910)	-	-
**FR23**	Glucose (SIGMA G8270)	Cottonseed flour (FLUKA C4898)	-
	Soluble starch from potato (PANREAC 121096)	-	-
	Cane molasses (Mercadona)	-	-
**M016**	Glucose (SIGMA G8270)	Bacto yeast extract (DIFCO 212750)	KH_2PO_4 (MERCK 1.04871)
	Soluble starch from potato (PANREAC 121096)	Bacto soytone (DIFCO 243620)	K_2HPO_4 (MERCK 5101)
	Maltose (MERCK 1.05910)	Bacto tryptone (DIFCO 211705)	$MgSO_4 \cdot 7H_2O$ (MERCK 5886)
	-	-	NaCl (MERCK 1.06404)
	-	-	$CaCl_2 \cdot 2H_2O$ (MERCK 1.02382)
	-	-	$SnCl_2 \cdot 2H_2O$; H_3BO_3; $Na_2MoO_4 \cdot 2H_2O$; $CuSO_4$;
	-	-	$CoCl_2 \cdot 6H_2O$; KCl; $ZnCl_2$; $MnSO_4 \cdot 4H_2O$; $FeCl_3$; HCl
**RAM2-P V2**	Glucose (SIGMA G8270)	Corn meal yellow (SIGMA C6304)	-
	Maltose (MERCK 1.05910)	Bacto yeast extract (DIFCO 212750)	-
	-	Proteose peptone (SIGMA P0431)	-
**SAM-6**	Glucose (SIGMA G8270)	Bacto yeast extract (DIFCO 212750)	$CoCl_2 \cdot 6H_2O$ (SIGMA C8661)
	Soluble starch from potato (PANREAC 121096)	NZ Amine type E (EKC) (SIGMA N4767)	$CaCO_3$ (ACROS ORGANICS 450680010)

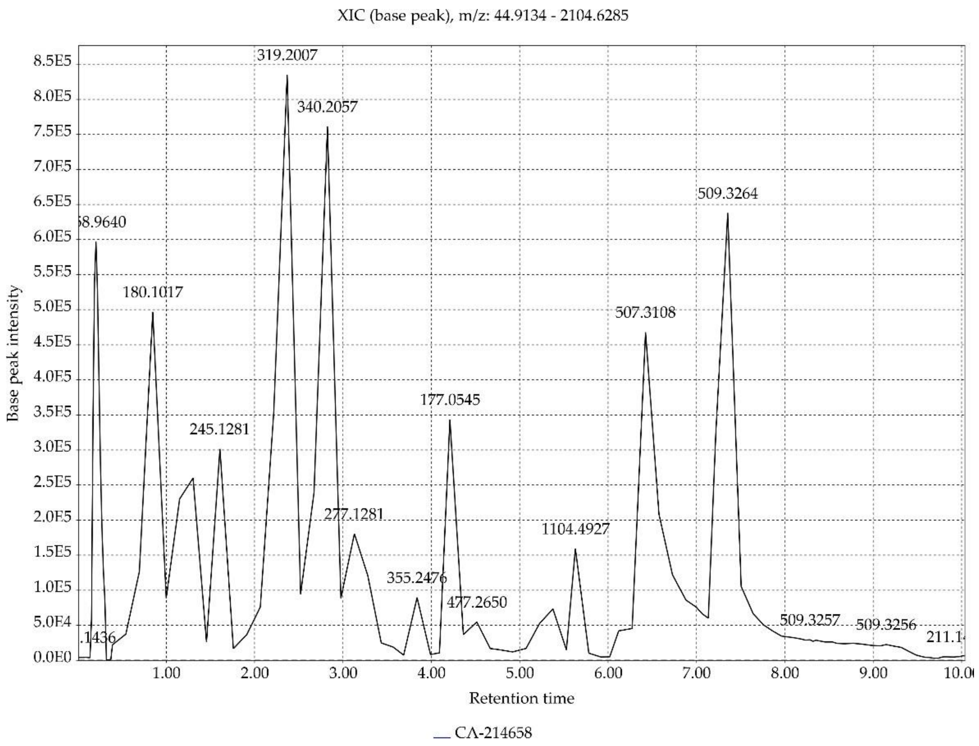

Figure S1. Total Ion Chromatogram (TIC) of the crude extract from the strain CA-214658 grown in RAM2-P V2 medium.

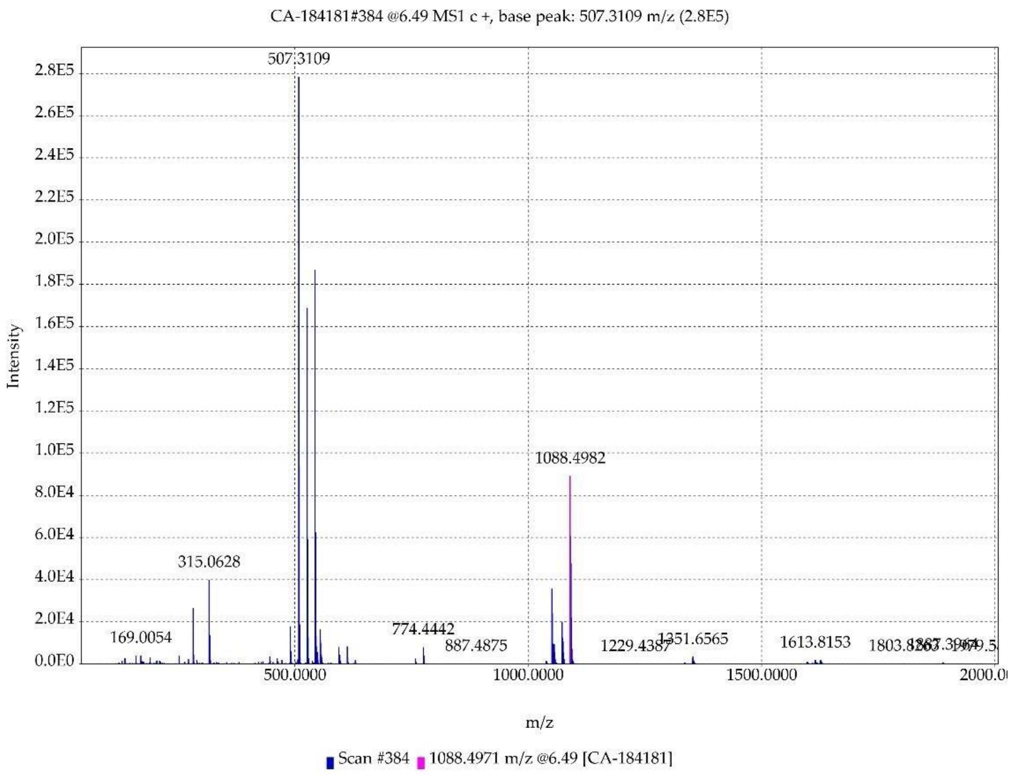

Figure S2. The ESI-TOF spectrum of phocoenamicin.

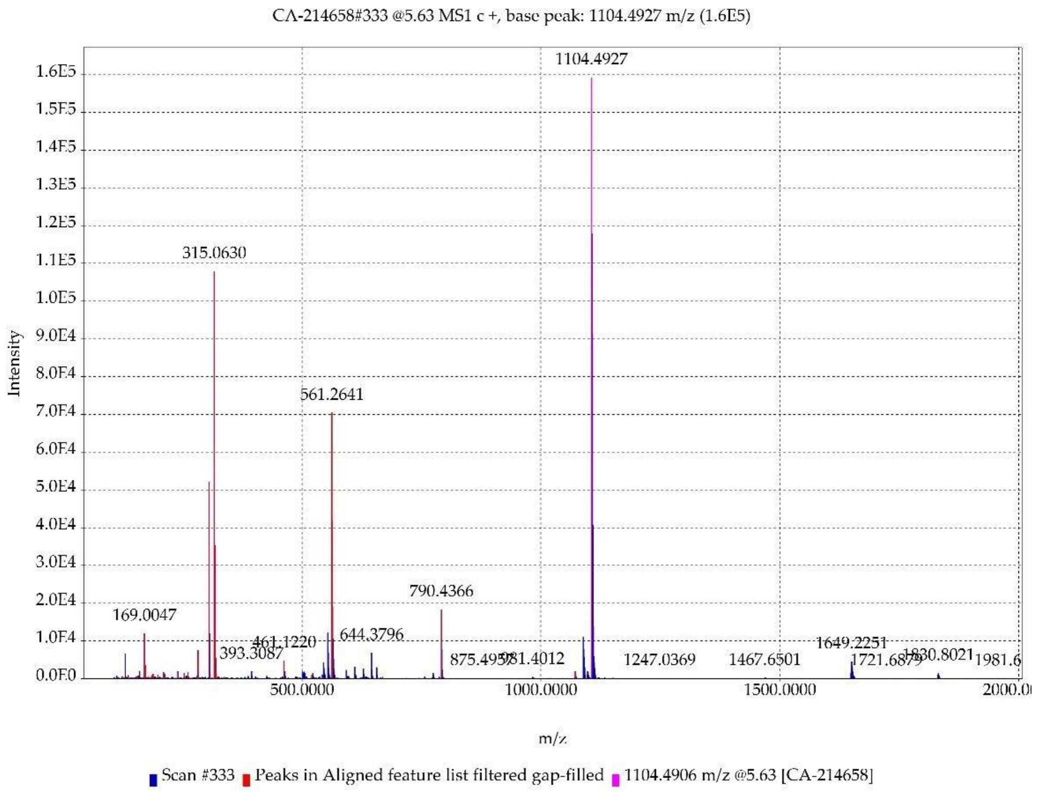

Figure S3. The ESI-TOF spectrum of phocoenamicin B.

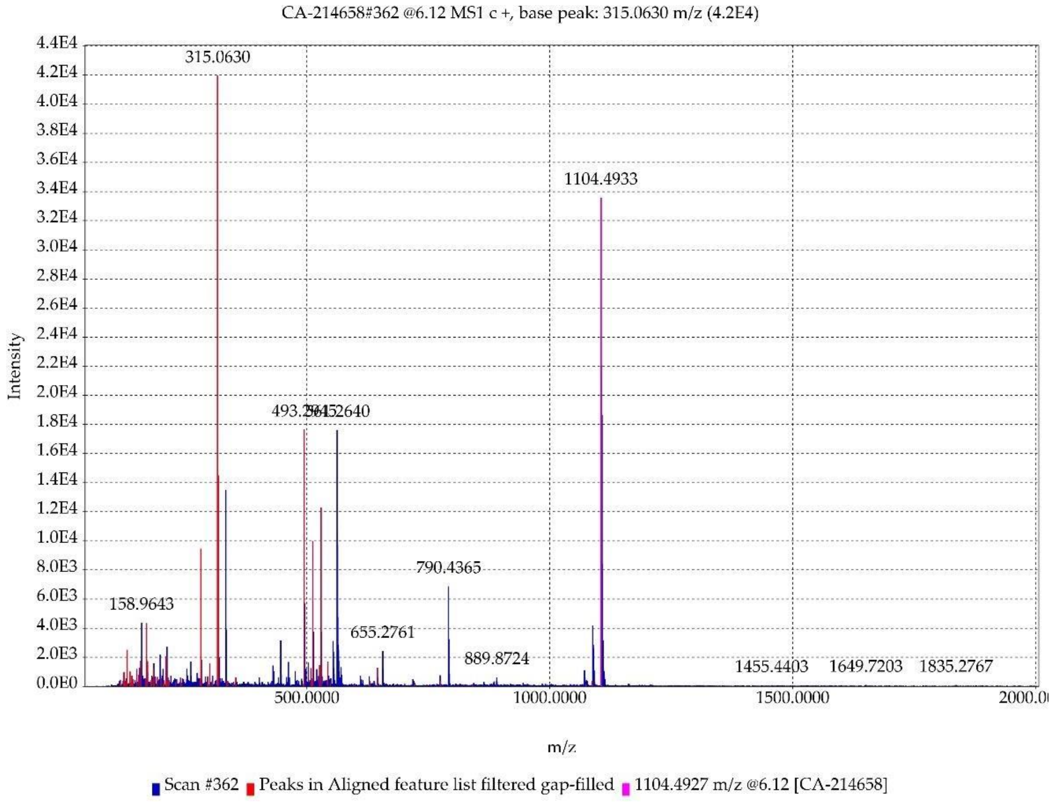

Figure S4. The ESI-TOF spectrum of phocoenamicin C.

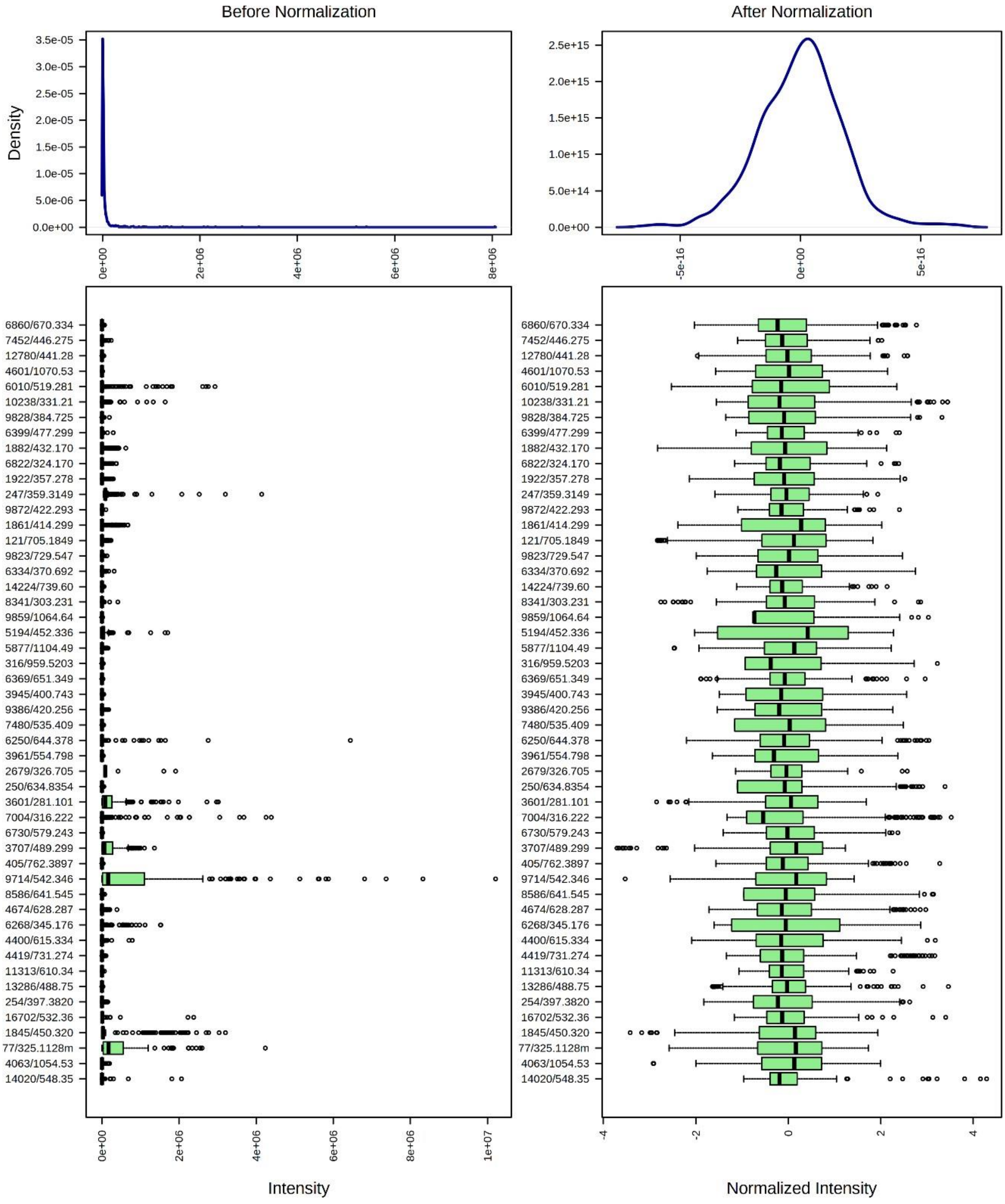

Figure S5. The boxplot and density plot showing the normalization result in MetaboAnalyst 5.0 after sample normalization, data transformation and scaling of the preprocessed LC-HRMS data obtained from the 270 extracts.

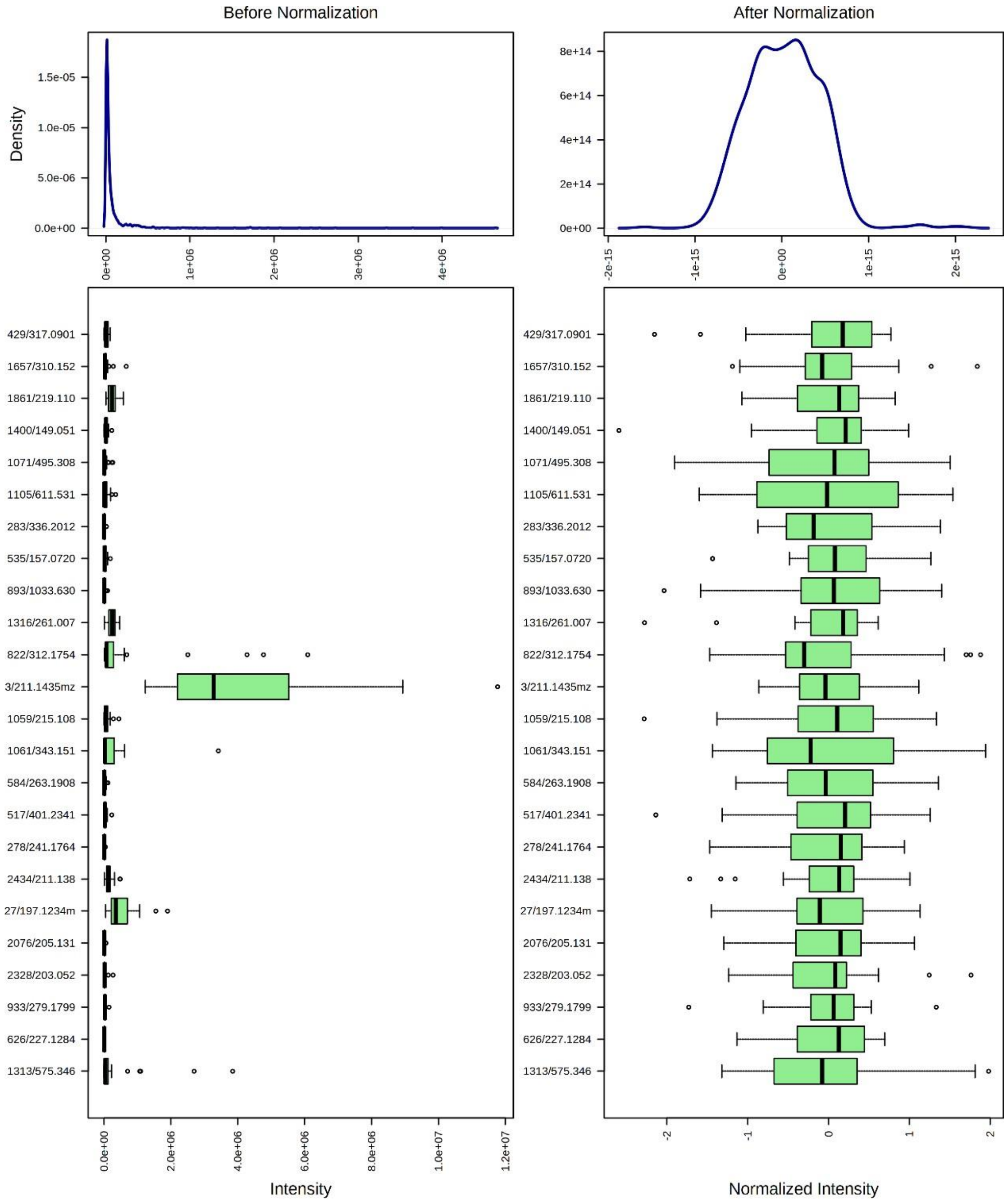

Figure S6. The boxplot and density plot showing the normalization result in MetaboAnalyst 5.0 after sample normalization, data transformation and scaling of the preprocessed LC-HRMS data obtained from the 27 extracts in RAM2-P V2 medium.

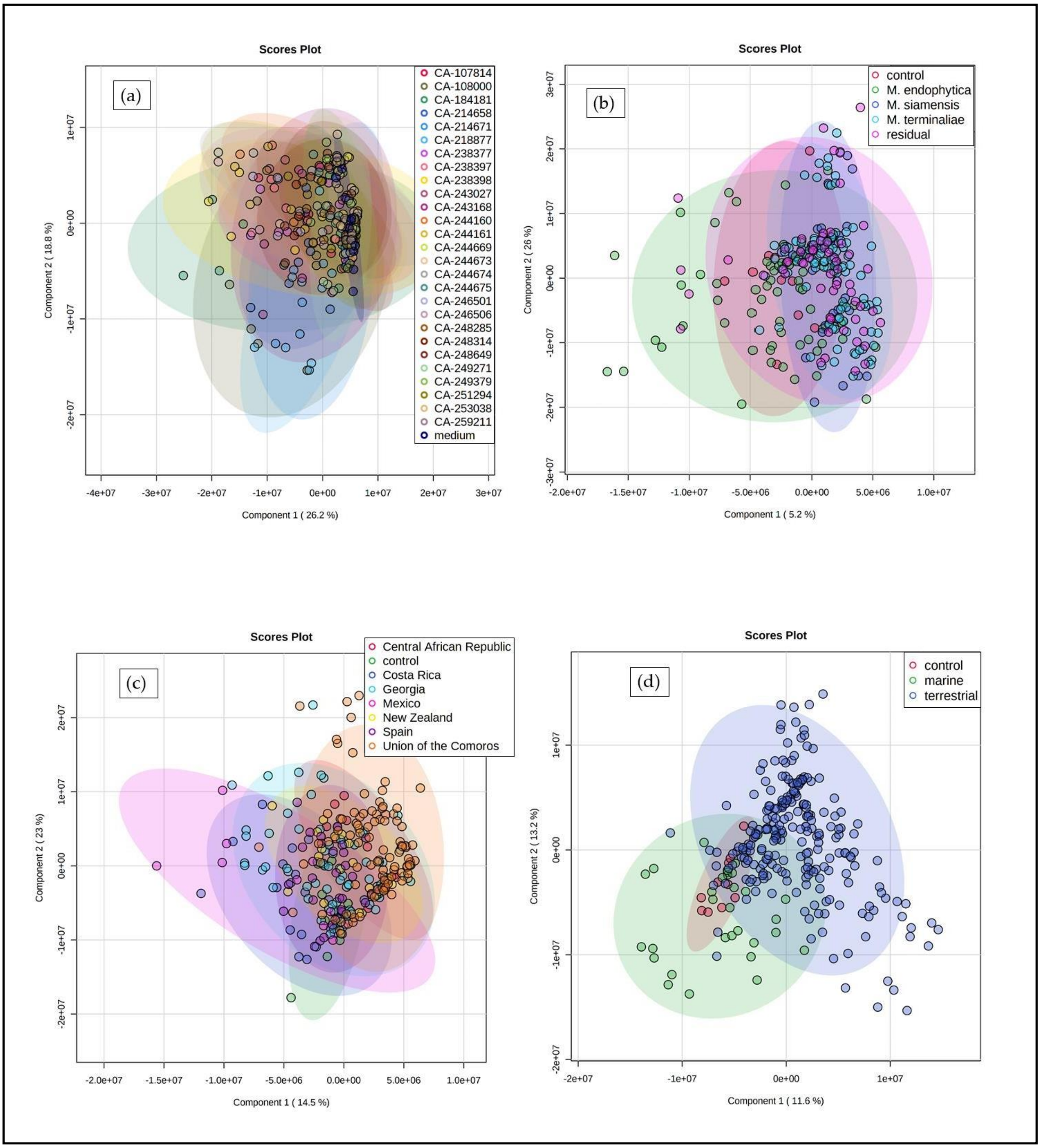

Figure S7. PLS-DA 2D Score plots of the 270 extracts for the parameters (a) different strains, (b) taxonomy species, (c) geographic origin and (d) ecology. All four models resulted in overfitting and lack of validation.

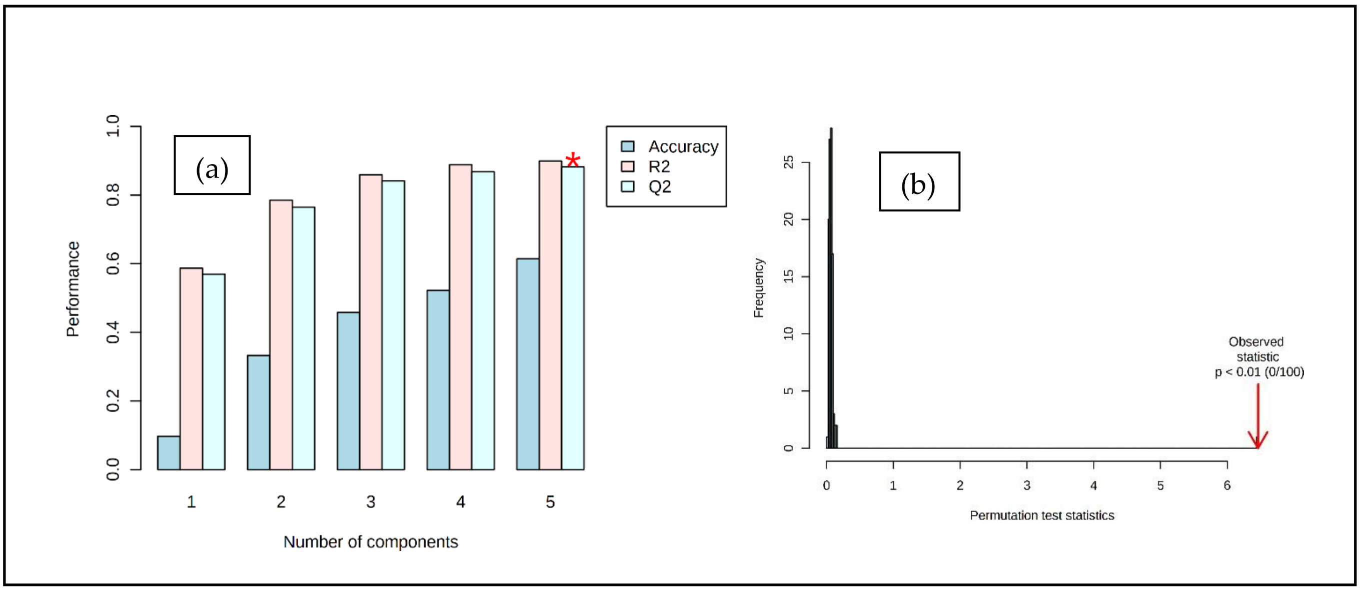

Figure S8. (a) Cross-validation test (CV) with values of R_2=0.859 and Q_2=0.841 and (b) permutation test with p-value < 0.01 of the PLS-DA analysis of the 270 extracts grouped by culture medium that validated its significance.

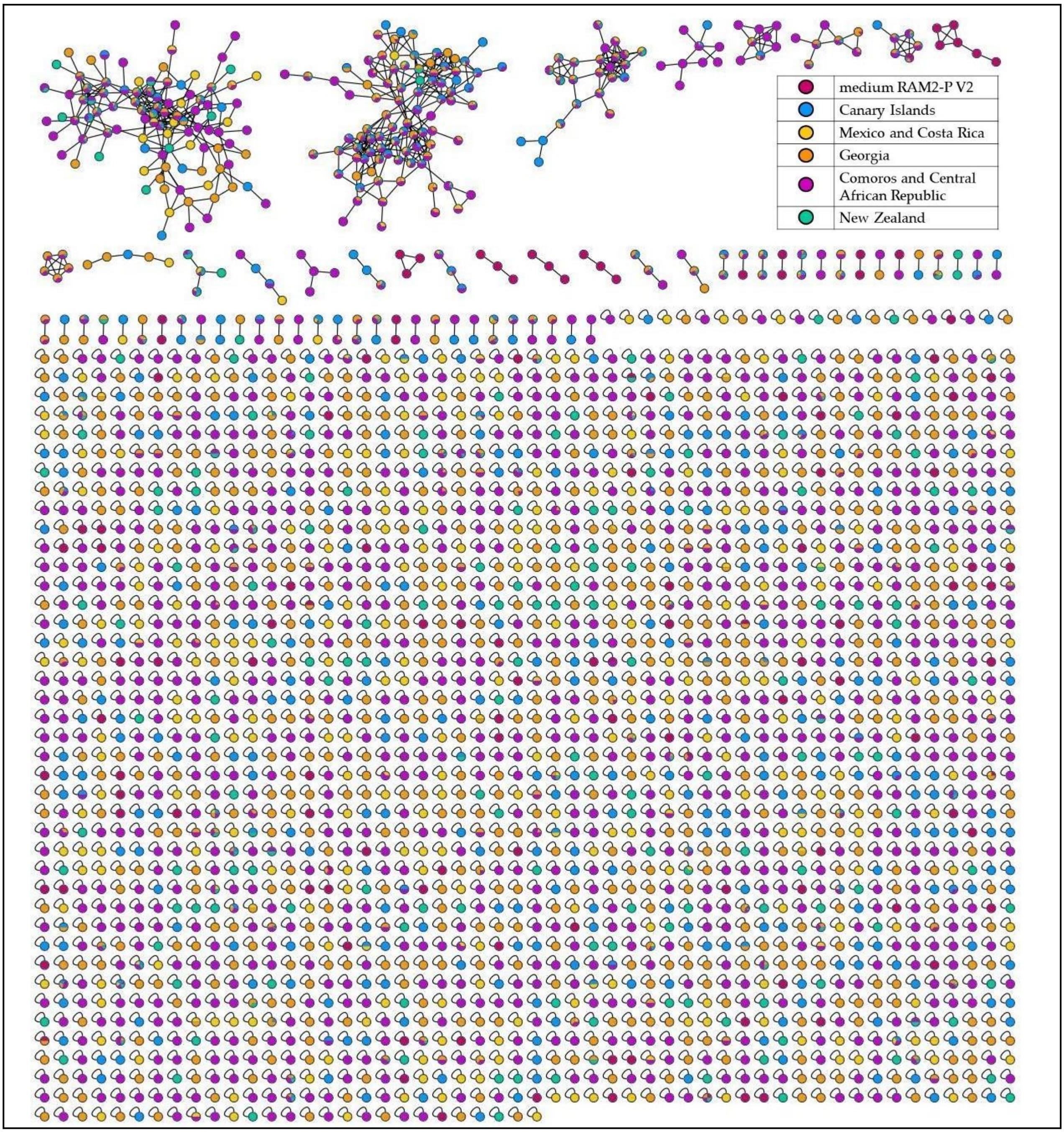

Figure S9. The classical molecular network (GNPS) generated from the extracts of the 27 strains cultured in RAM2-P V2 medium. The nodes are coloured according to the geographic origin of the 27 strains.

Table S2. Average % inhibition of the 270 extracts against MRSA MB5393, *M. bovis* BCG and *M. tuberculosis* H37Ra.

		Average % Inhibition		
**STRAIN**	**CULTURE MEDIUM**	**MRSA MB5393**	***M. tuberculosis* H37Ra**	***M. bovis* BCG**
CA-107814	APM9	-47,2	-89,7	-88,5
CA-107814	DEF-15	36,6	-38,9	-7,9
CA-107814	DEF-15S	25,8	-50,7	-10,2
CA-107814	DNPM	5,5	-96,8	-93,9
CA-107814	FPY-12	4,1	-86,9	-78,9
CA-107814	FPY-2	-99,1	-90,5	-89,1
CA-107814	FR23	-51,8	-93,8	-91,7
CA-107814	M016	-5,5	-90,5	-89,7
CA-107814	RAM2-P V2	-94,7	-92,1	-92,2
CA-107814	SAM-6	-4,5	-95,8	-93,5
CA-108000	APM9	-99,8	-90,0	-88,3
CA-108000	DEF-15	37,5	-75,6	-9,9
CA-108000	DEF-15S	28,2	-91,8	-11,7
CA-108000	DNPM	20,4	-97,0	-94,1
CA-108000	FPY-12	-97,0	-88,1	-88,1
CA-108000	FPY-2	-99,4	-90,6	-88,7
CA-108000	FR23	-38,1	-93,4	-90,7
CA-108000	M016	-99,5	-93,2	-91,8
CA-108000	RAM2-P V2	-90,4	-96,8	-94,5
CA-108000	SAM-6	-63,5	-95,2	-93,1
CA-184181	APM9	-94,7	-94,1	-92,2
CA-184181	DEF-15	23,8	-95,6	-9,8
CA-184181	DEF-15S	4,2	-60,6	-90,1
CA-184181	DNPM	25,1	-96,9	-91,4
CA-184181	FPY-12	-79,6	-87,7	-87,6
CA-184181	FPY-2	-101,7	-93,4	-91,6
CA-184181	FR23	-98,9	-96,9	-94,0
CA-184181	M016	-98,7	-97,3	-94,5
CA-184181	RAM2-P V2	-72,2	-97,1	-94,7
CA-184181	SAM-6	-100,9	-96,0	-93,2
CA-214658	APM9	-75,7	-91,7	-89,8
CA-214658	DEF-15	40,3	-35,5	-7,3
CA-214658	DEF-15S	27,2	-90,0	-23,4
CA-214658	DNPM	25,1	-96,5	-92,5
CA-214658	FPY-12	-36,3	-89,3	-88,3
CA-214658	FPY-2	-100,0	-93,0	-90,7
CA-214658	FR23	-65,7	-95,5	-91,7
CA-214658	M016	-101,9	-92,9	-91,5
CA-214658	RAM2-P V2	-67,6	-94,4	-92,5
CA-214658	SAM-6	5,9	-95,0	-90,5
CA-214671	APM9	-101,0	-89,4	-88,0

CA-214671	DEF-15	37,3	-62,5	-6,7
CA-214671	DEF-15S	26,5	-94,6	-50,9
CA-214671	DNPM	-16,0	-97,1	-93,5
CA-214671	FPY-12	-92,4	-92,2	-90,1
CA-214671	FPY-2	-92,3	-93,8	-91,7
CA-214671	FR23	16,8	-93,4	-28,7
CA-214671	M016	-90,0	-92,3	-89,6
CA-214671	RAM2-P V2	-68,8	-94,6	-91,9
CA-214671	SAM-6	-21,4	-57,9	-92,0
CA-218877	APM9	-97,0	-90,7	-86,5
CA-218877	DEF-15	39,2	-96,5	-11,5
CA-218877	DEF-15S	28,0	-88,9	-23,8
CA-218877	DNPM	-46,6	-96,5	-93,6
CA-218877	FPY-12	12,1	-88,3	-60,5
CA-218877	FPY-2	-86,7	-92,8	-90,7
CA-218877	FR23	9,8	-77,5	-84,8
CA-218877	M016	-101,5	-65,8	-15,1
CA-218877	RAM2-P V2	-77,3	-96,1	-93,0
CA-218877	SAM-6	15,8	-97,0	-93,0
CA-238377	APM9	-92,5	-92,9	-90,6
CA-238377	DEF-15	37,8	-26,7	-5,6
CA-238377	DEF-15S	5,8	-61,6	-32,1
CA-238377	DNPM	-98,0	-66,0	-94,1
CA-238377	FPY-12	-100,5	-89,6	-88,1
CA-238377	FPY-2	-94,0	-91,8	-88,6
CA-238377	FR23	-97,4	-31,4	-94,2
CA-238377	M016	-99,9	-97,0	-94,7
CA-238377	RAM2-P V2	-91,2	-96,5	-92,9
CA-238377	SAM-6	-99,7	-28,7	-72,2
CA-238397	APM9	-99,7	-91,8	-90,4
CA-238397	DEF-15	40,4	-69,5	-6,3
CA-238397	DEF-15S	23,2	-11,7	-7,0
CA-238397	DNPM	28,9	-94,4	-15,9
CA-238397	FPY-12	31,3	-6,0	-18,5
CA-238397	FPY-2	-98,8	-89,2	-88,7
CA-238397	FR23	-149,1	-34,0	-58,2
CA-238397	M016	-96,0	-72,0	-92,7
CA-238397	RAM2-P V2	-95,5	-92,9	-90,0
CA-238397	SAM-6	-100,2	-2,3	-60,8
CA-238398	APM9	-38,4	-92,4	-86,7
CA-238398	DEF-15	39,9	-7,5	-5,0
CA-238398	DEF-15S	27,0	-4,6	-5,0
CA-238398	DNPM	-3,1	-97,2	-41,5
CA-238398	FPY-12	-44,9	-10,0	-13,4
CA-238398	FPY-2	-55,0	-94,6	-52,7
CA-238398	FR23	-62,1	-95,2	-91,8
CA-238398	M016	-99,8	-93,5	-89,9

CA-238398	RAM2-P V2	0,1	-95,6	-68,3
CA-238398	SAM-6	-101,1	-95,8	-92,6
CA-243027	APM9	-88,9	-92,8	-89,7
CA-243027	DEF-15	38,7	-90,1	-10,1
CA-243027	DEF-15S	28,3	-11,1	-10,8
CA-243027	DNPM	33,7	-96,3	-93,2
CA-243027	FPY-12	28,3	-6,1	-9,4
CA-243027	FPY-2	-49,4	-90,4	-88,3
CA-243027	FR23	-97,1	-92,1	-93,8
CA-243027	M016	-79,7	-97,1	-94,8
CA-243027	RAM2-P V2	-62,6	-93,6	-89,3
CA-243027	SAM-6	-64,4	-95,4	-92,4
CA-243168	APM9	-79,9	-87,2	-89,8
CA-243168	DEF-15	41,6	-35,3	-7,2
CA-243168	DEF-15S	30,1	-97,6	-14,6
CA-243168	DNPM	14,5	-96,3	-86,1
CA-243168	FPY-12	-99,9	-87,7	-87,2
CA-243168	FPY-2	-89,7	-91,4	-89,1
CA-243168	FR23	38,8	-94,5	-39,4
CA-243168	M016	-97,1	-94,7	-92,4
CA-243168	RAM2-P V2	-100,4	-95,5	-91,9
CA-243168	SAM-6	-59,4	-96,2	-92,5
CA-244160	APM9	-95,7	-90,5	-88,5
CA-244160	DEF-15	40,2	4,3	-2,2
CA-244160	DEF-15S	4,8	-94,8	-84,7
CA-244160	DNPM	29,1	-98,7	-92,1
CA-244160	FPY-12	-115,7	-89,1	-86,5
CA-244160	FPY-2	-35,3	-91,9	-90,1
CA-244160	FR23	-54,8	-96,4	-92,5
CA-244160	M016	-100,2	-95,7	-94,0
CA-244160	RAM2-P V2	-53,9	-95,2	-92,4
CA-244160	SAM-6	-102,4	-93,6	-91,8
CA-244161	APM9	-87,9	-89,9	-89,1
CA-244161	DEF-15	37,5	-97,7	-22,6
CA-244161	DEF-15S	5,2	-97,9	-21,4
CA-244161	DNPM	18,1	-98,8	-92,4
CA-244161	FPY-12	-94,6	-89,4	-88,2
CA-244161	FPY-2	-95,2	-90,3	-89,7
CA-244161	FR23	-18,7	-96,9	-95,4
CA-244161	M016	-99,8	-94,9	-91,8
CA-244161	RAM2-P V2	-98,6	-96,3	-92,9
CA-244161	SAM-6	-100,4	-95,0	-92,3
CA-244669	APM9	-9,7	-90,5	-73,1
CA-244669	DEF-15	-7,1	-10,2	-4,6
CA-244669	DEF-15S	-23,1	-6,4	-5,2
CA-244669	DNPM	20,6	4,9	-10,3
CA-244669	FPY-12	-48,1	-88,3	-15,8

CA-244669	FPY-2	31,9	-62,7	-6,3
CA-244669	FR23	-99,4	-95,7	-91,1
CA-244669	M016	-99,9	-94,9	-90,6
CA-244669	RAM2-P V2	6,4	-78,4	-14,1
CA-244669	SAM-6	-99,5	-74,3	-92,7
CA-244673	APM9	-82,0	-94,0	-91,0
CA-244673	DEF-15	29,5	-98,8	-55,8
CA-244673	DEF-15S	24,1	-82,8	-69,4
CA-244673	DNPM	24,4	-17,4	-8,9
CA-244673	FPY-12	-82,8	-88,0	-86,5
CA-244673	FPY-2	24,2	-91,5	-88,4
CA-244673	FR23	-94,5	-96,2	-92,0
CA-244673	M016	-157,3	-33,7	-57,6
CA-244673	RAM2-P V2	34,8	-93,5	-86,7
CA-244673	SAM-6	-99,4	-94,9	-90,4
CA-244674	APM9	-90,4	-91,1	-89,7
CA-244674	DEF-15	36,6	2,5	-0,4
CA-244674	DEF-15S	10,7	-97,4	-65,7
CA-244674	DNPM	15,7	-97,5	-13,8
CA-244674	FPY-12	-90,6	-89,6	-87,9
CA-244674	FPY-2	37,9	-61,7	-9,1
CA-244674	FR23	-86,6	-42,6	-83,4
CA-244674	M016	-87,0	-84,6	-96,0
CA-244674	RAM2-P V2	-99,0	-95,7	-93,3
CA-244674	SAM-6	-85,0	-94,3	-90,3
CA-244675	APM9	-90,0	-90,4	-88,0
CA-244675	DEF-15	22,3	-96,7	-4,7
CA-244675	DEF-15S	-3,8	-98,0	-62,1
CA-244675	DNPM	-98,6	-92,2	-93,9
CA-244675	FPY-12	-98,0	-88,7	-87,7
CA-244675	FPY-2	-81,5	-92,2	-90,5
CA-244675	FR23	-126,8	-81,8	-92,7
CA-244675	M016	-99,5	-96,0	-91,9
CA-244675	RAM2-P V2	-99,3	-94,4	-90,8
CA-244675	SAM-6	-98,9	-95,4	-84,8
CA-246501	APM9	-91,9	-94,1	-91,1
CA-246501	DEF-15	42,0	-0,7	-2,6
CA-246501	DEF-15S	8,6	-62,3	-81,3
CA-246501	DNPM	-41,9	-98,0	-93,8
CA-246501	FPY-12	-139,0	-89,4	-88,4
CA-246501	FPY-2	-91,5	-91,5	-88,5
CA-246501	FR23	-95,6	-97,1	-76,1
CA-246501	M016	-98,6	-96,4	-91,6
CA-246501	RAM2-P V2	-103,9	-95,2	-92,4
CA-246501	SAM-6	-100,3	-96,4	-92,9
CA-246506	APM9	-99,5	-94,8	-92,0
CA-246506	DEF-15	18,6	-96,3	-8,6

CA-246506	DEF-15S	7,1	-96,8	-73,5
CA-246506	DNPM	-1,3	-95,0	-25,9
CA-246506	FPY-12	-57,3	-90,0	-82,0
CA-246506	FPY-2	-85,8	-92,8	-89,8
CA-246506	FR23	-98,1	-96,9	-93,0
CA-246506	M016	-99,3	-96,4	-93,7
CA-246506	RAM2-P V2	-88,7	-96,8	-93,8
CA-246506	SAM-6	-103,3	-95,8	-92,9
CA-248285	APM9	37,1	-93,1	-82,0
CA-248285	DEF-15	39,1	-97,0	-37,3
CA-248285	DEF-15S	18,5	-95,5	-5,5
CA-248285	DNPM	5,9	-97,7	-92,9
CA-248285	FPY-12	-74,5	-87,0	-86,3
CA-248285	FPY-2	-99,5	-88,2	-87,1
CA-248285	FR23	-108,3	-45,9	-47,4
CA-248285	M016	-98,2	-99,3	-96,0
CA-248285	RAM2-P V2	-98,5	-94,3	-91,1
CA-248285	SAM-6	-132,8	-93,6	-91,1
CA-248314	APM9	-94,6	-90,6	-86,7
CA-248314	DEF-15	43,6	-94,2	-11,5
CA-248314	DEF-15S	33,6	-93,9	-20,6
CA-248314	DNPM	44,7	-97,3	-92,5
CA-248314	FPY-12	-45,1	-87,8	-77,1
CA-248314	FPY-2	-97,3	-89,0	-88,6
CA-248314	FR23	-86,5	-93,9	-90,7
CA-248314	M016	-116,9	-93,0	-90,3
CA-248314	RAM2-P V2	-85,5	-93,4	-91,1
CA-248314	SAM-6	-12,3	-94,7	-92,5
CA-248649	APM9	-95,8	-87,5	-63,7
CA-248649	DEF-15	24,6	-46,2	-3,3
CA-248649	DEF-15S	4,2	-98,7	-25,0
CA-248649	DNPM	24,7	-98,2	-13,9
CA-248649	FPY-12	-98,8	-89,4	-86,5
CA-248649	FPY-2	-81,3	-90,2	-87,7
CA-248649	FR23	-83,1	-98,5	-77,3
CA-248649	M016	-101,0	-95,2	-90,1
CA-248649	RAM2-P V2	-100,1	-92,1	-90,8
CA-248649	SAM-6	-99,7	-95,5	-92,0
CA-249271	APM9	24,9	-91,2	-40,5
CA-249271	DEF-15	44,1	-95,3	-9,4
CA-249271	DEF-15S	26,5	-98,6	-24,1
CA-249271	DNPM	25,1	-96,5	-80,2
CA-249271	FPY-12	-98,8	-89,7	-85,6
CA-249271	FPY-2	-68,4	-93,7	-91,1
CA-249271	FR23	33,4	-94,9	-33,8
CA-249271	M016	-100,2	-94,1	-90,9
CA-249271	RAM2-P V2	-84,4	-95,4	-92,4

CA-249271	SAM-6	-82,5	-95,8	-89,4
CA-249379	APM9	-80,5	-93,2	-90,4
CA-249379	DEF-15	42,9	-75,0	-3,1
CA-249379	DEF-15S	15,9	-99,0	-49,8
CA-249379	DNPM	-99,7	-98,1	-93,1
CA-249379	FPY-12	-59,8	-89,8	-76,1
CA-249379	FPY-2	13,9	-92,8	-90,8
CA-249379	FR23	-32,3	-94,3	-90,4
CA-249379	M016	-99,6	-98,1	-94,5
CA-249379	RAM2-P V2	-97,9	-94,3	-88,5
CA-249379	SAM-6	-85,6	-84,8	-90,6
CA-251294	APM9	-76,9	-93,6	-90,5
CA-251294	DEF-15	23,3	-93,6	-22,1
CA-251294	DEF-15S	2,3	-96,9	-28,6
CA-251294	DNPM	-79,1	-97,8	-93,5
CA-251294	FPY-12	-99,9	-87,5	-87,0
CA-251294	FPY-2	-149,5	-94,4	-90,9
CA-251294	FR23	-53,5	-97,8	-91,3
CA-251294	M016	-89,9	-95,2	-91,8
CA-251294	RAM2-P V2	-98,4	-96,1	-90,6
CA-251294	SAM-6	-99,1	-95,1	-91,6
CA-253038	APM9	-89,1	-93,8	-90,3
CA-253038	DEF-15	27,1	-98,0	-30,9
CA-253038	DEF-15S	7,6	-70,9	-5,1
CA-253038	DNPM	-38,0	-99,0	-76,6
CA-253038	FPY-12	-95,9	-90,0	-86,6
CA-253038	FPY-2	1,7	-94,9	-87,0
CA-253038	FR23	-118,0	-34,4	-29,4
CA-253038	M016	-72,1	-27,7	-31,4
CA-253038	RAM2-P V2	-94,7	-99,0	-94,9
CA-253038	SAM-6	-100,2	-89,0	-89,7
CA-259211	APM9	-41,6	-91,9	-88,8
CA-259211	DEF-15	41,2	-93,7	-14,2
CA-259211	DEF-15S	42,3	-97,3	-35,1
CA-259211	DNPM	-101,3	-96,1	-4,5
CA-259211	FPY-12	36,9	-87,2	-55,4
CA-259211	FPY-2	-100,8	-92,0	-87,7
CA-259211	FR23	-84,7	-98,5	-96,2
CA-259211	M016	-98,8	-97,5	-94,4
CA-259211	RAM2-P V2	-100,8	-94,5	-90,7
CA-259211	SAM-6	-109,0	-94,9	-92,6
